# A Case Report of Crescentic Glomerulonephritis With Positive Serum Anti-glomerular Basement Membrane Without Linear Glomerular Basement Membrane Immunofluorescent Staining

**DOI:** 10.7759/cureus.24879

**Published:** 2022-05-10

**Authors:** Mohannad Faisal, Abdullah Shams, Suresh Archichige, Ahmed Hamdi, Mohammed Akhtar

**Affiliations:** 1 Internal Medicine, Hamad Medical Corporation, Doha, QAT; 2 internal Medicine, Hamad Medical Corporation, Doha, QAT; 3 Nephrology, Hamad Medical Corporation, Doha, QAT; 4 Laboratory Medicine and Pathology, Hamad Medical Corporation, Hamad General Hospital, Doha, QAT

**Keywords:** acute glomerulonephritis, necrotizing and crescentic glomerulonephritis, glomerular disorders, acute renal injury, anti-gbm

## Abstract

Anti-glomerular basement membrane (anti-GBM) disease is an autoimmune disorder characterized by the production of circulating immunoglobulin G (IgG) antibodies that affect the kidneys and lungs, mainly in the form of rapidly progressive crescentic glomerulonephritis and pulmonary hemorrhage. Typically diagnosed on tissue biopsy, findings mainly include glomerular crescent formation, bright linear staining of GBM for IgG on direct immunofluorescence (IF), and the serologic presence of circulating anti-GBM antibodies. Variation in the laboratory results, where histological findings of linear IgG IF staining were present in the absence of circulating anti-GBM antibodies, have recently led to the use of the term “atypical anti-GBM disease,” which usually has a distinct benign clinical outcome as compared to typical anti-GBM disease. We report a case of a middle-aged woman who presented with renal failure without lung involvement. Upon further investigation, the patient was found to have strongly positive serum anti-GBM antibodies, but the tissue biopsy did not show typical findings of the anti-GBM disease. The patient showed modest improvement after multiple sessions of plasmapheresis and steroids, with stabilization of her renal parameters after the initial response. In our case, we will address the possibilities of the discrepancies between the serological and histopathological findings.

## Introduction

Typical anti-glomerular basement membrane (anti-GBM) disease involves the lungs and kidneys along with the production of circulating anti-GBM immunoglobulin G (IgG) antibodies and a histological pattern that has linear basement membrane staining on immunofluorescence (IF), ideally performed on a fresh kidney biopsy sample. On the contrary, the atypical anti-GBM disease has been regarded as a variant where histological evidence is present in the absence of the circulating anti-GBM IgG antibodies, resulting in a benign overall outcome as compared to the typical disease. There have been other rare variants reported in the literature where circulating IgG anti-GBM antibodies are present, causing rapidly progressive crescentic glomerulonephritis in the absence of typical histology findings on kidney biopsy, sparing the lungs. To the best of our knowledge, there have been four cases reported so far [1], and we are presenting a similar case, which is the first from the state of Qatar.

## Case presentation

Our case is a 61-year-old Indian lady, known to have hypertension on amlodipine and perindopril, who presented to the hospital with about 10 days history of abdominal pain. The pain was 5/10 in intensity and dull in nature. The pain was constant but worse at night. There was no radiation of the pain. There was no associated nausea or vomiting. There was a 20-day history of dark urine. She complained of decreased appetite since the passing of her husband a few months prior to presentation. The patient denied prolonged exposure to the sun. She was not taking any new medications or herbal supplements. There was no recent use of non-steroidal anti-inflammatory drugs or other pain medications. There were no reported recent infections, bone or joint pains, rashes, or skin changes. Her past surgical history was only remarkable for cholecystectomy done about four years ago. She worked in a private home as a cook. She was married with two children. She denied smoking and alcohol use. There were no known allergies to food or medications. No known family history of kidney disease or other conditions was reported.

On presentation to the emergency department, she was afebrile with a respiratory rate of 17 breaths per minute, heart rate of 57 beats per minute, and blood pressure of 137/76 mmHg. On general examination, there was good skin turgor, wet oral cavities, and good capillary refill. The abdomen was soft and lax with no organomegaly or loin tenderness. Examination of the musculoskeletal system revealed no joint tenderness or swelling. There were no skin changes or rashes.

**Figure 1:**
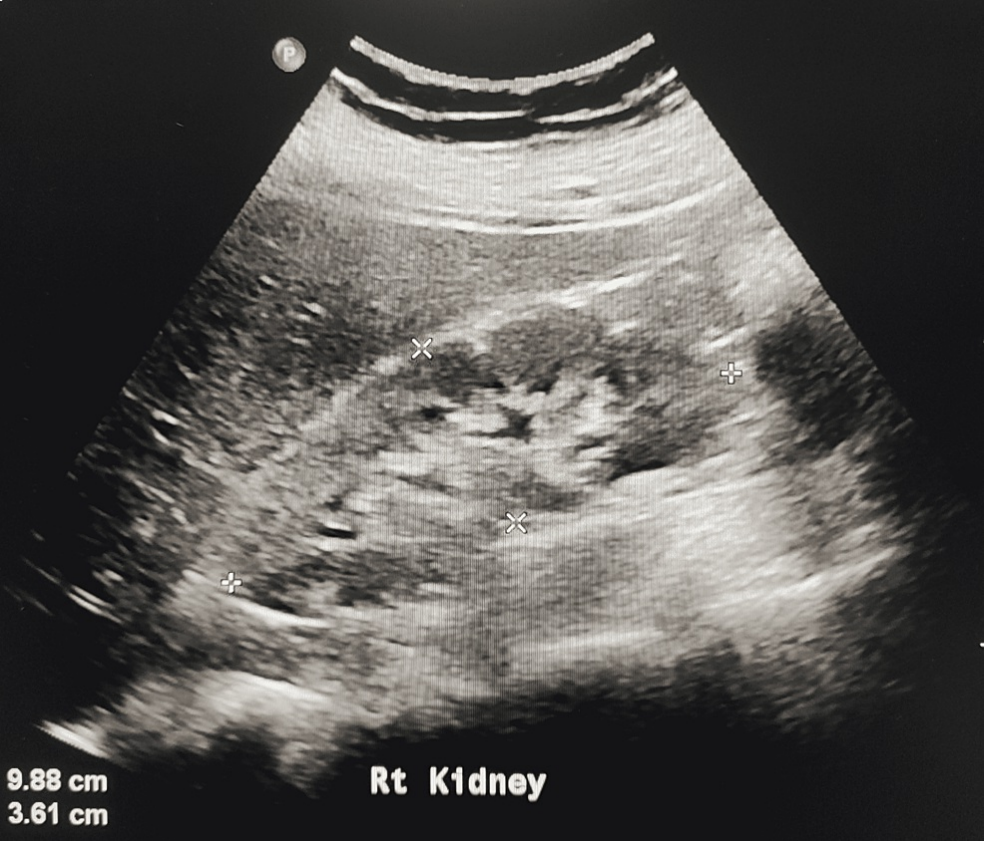
Ultrasound of the right kidney showing increased cortical echogenicity

**Figure 2:**
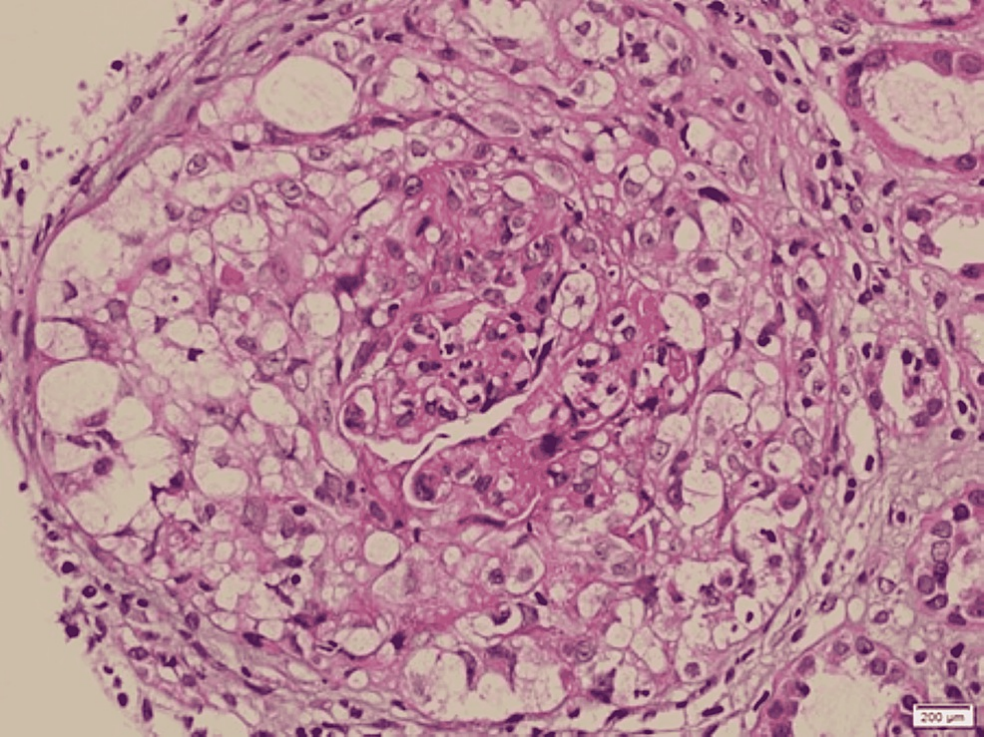
Light microscopic examination of crescentic glomerulonephritis with hematoxylin-eosin stain A circumferential cellular crescent with segmental fibrinoid necrosis x40 times magnification

**Figure 3:**
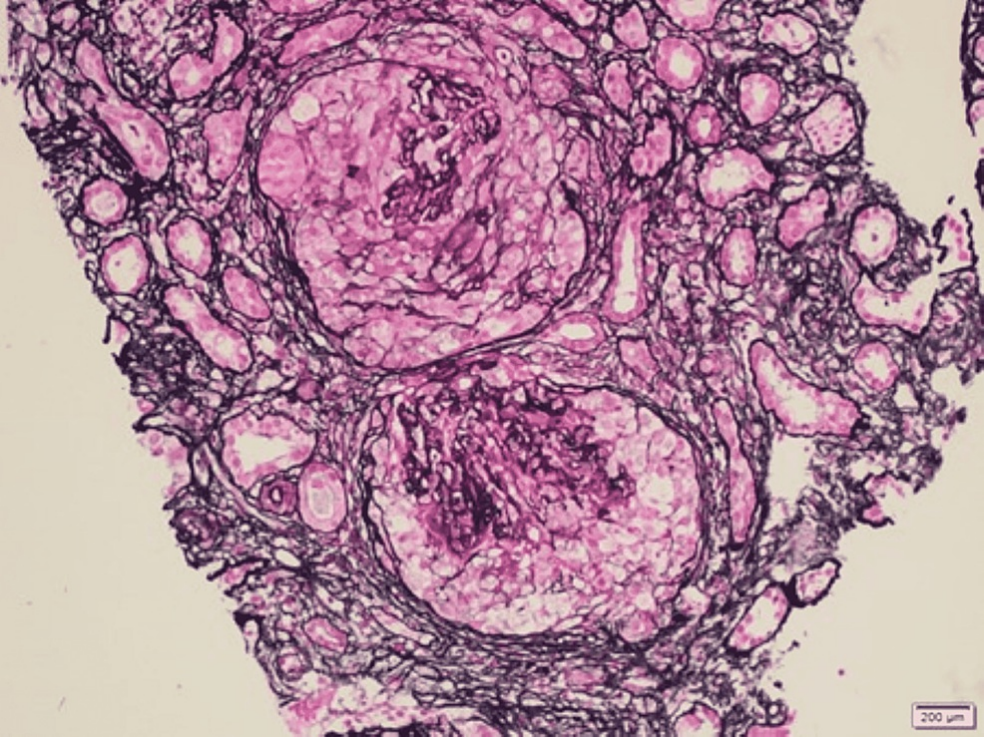
Light microscopic examination of crescentic glomerulonephritis with Jones silver staining Two glomeruli with residual glomerular tufts surrounded by circumferential cellular crescents. One glomerulus shows early focal disruption of the Bowman’s capsule. x20 times magnification

**Figure 4:**
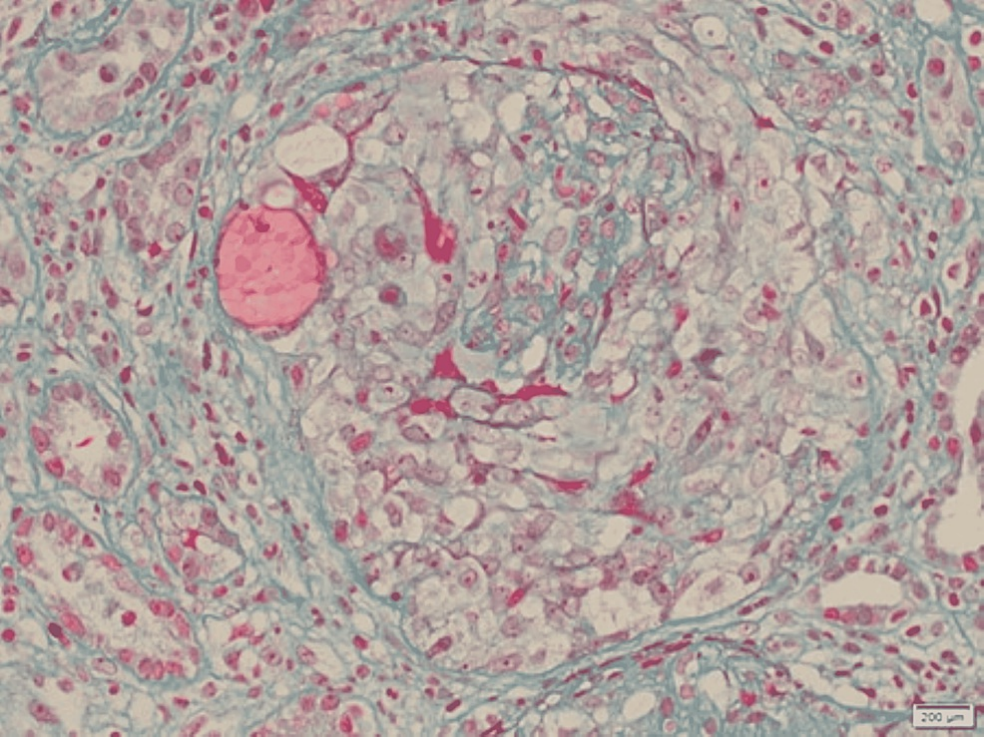
Examination under light microscopy using Masson trichrome stain Segmental areas of fibrinoid necrosis in the glomerular tuft are highlighted in red with focal disruption in the Bowman’s capsule. x40 times magnification

**Figure 5:**
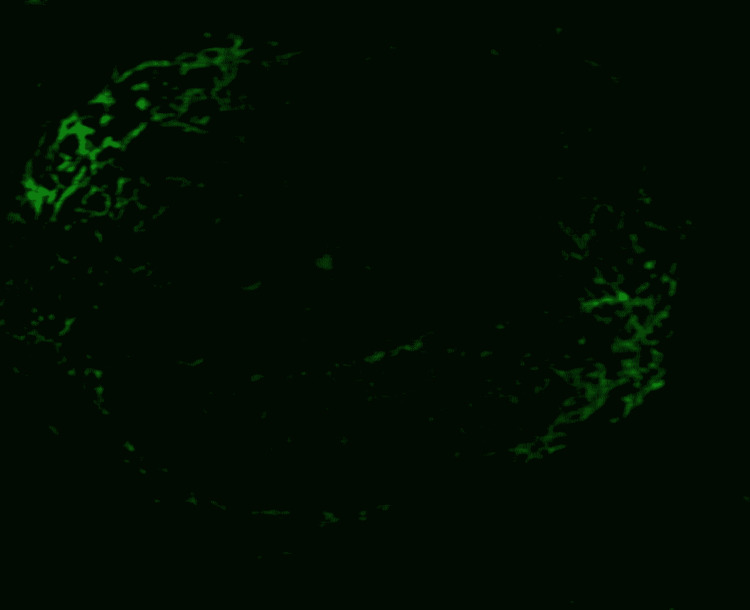
Examination under immunofluorescence microscopy Negative linear immunoglobulin G (IgG) staining of the glomerular basement membrane

The initial labs (Table 1) showed a normal complete blood count. The serum creatinine (Cr) on admission was elevated at 467 µmol/L. However, her creatinine one month prior to admission was 165 µmol/L and was normal at 88 µmol/L about one year prior to admission. The urine dipstick was negative for leukocytes but showed proteinuria and hematuria. Further investigation showed elevated parathyroid hormone, phosphate, and urine protein/Cr ratio. The autoimmune workup showed positive anti-nuclear antibody (ANA), anti-GBM antibodies with a titer of 206 U/ml, along with positive anti-Ro antibodies. The rest of the immune workup, including the anti-myeloperoxidase and antibody against proteinase-3 (PR3) were negative (Table 2). The complement (C3, C4) levels were normal. Workup for an infectious cause yielded negative results for human immunodeficiency virus, hepatitis C, and hepatitis B. Furthermore, the multiple myeloma workup was negative. Ultrasound abdomen showed normal-sized kidneys with increased parenchymal echogenicity as shown in Figure 1. No features of obstructive uropathy were noted. A kidney biopsy had been done on paraffin-embedded material and showed features of crescentic glomerulonephritis (Figures 2-4). However, the immunofluorescent staining showed no corresponding linear IgG pattern (Figure 5).

**Table 1:** Complete blood count (CBC) and complete metabolic panel (CMP) at admission µL- Micro liters; gm/dL- grams/deciliter; mmol/L – millimoles per liter; µmol/L – micromole per liter; mg/L – milligrams per liter

Test Type	Value	Normal Range
WBC (x 10^3^/µl)	9.9	4-10
Neutrophil (%)	68.1	55-70
Lymphocyte (%)	19.0	20 - 40
Monocyte (%)	10.0	2 - 8
Eosinophil (%)	2.4	1 - 4
Basophil (%)	0.5	0.5 - 1
Hb (gm/dL)	9.1	12-15
Platelets (x103/µL)	302	150-400
Urea (mmol/L)	18.6	2.5 – 6.7
Cr (µmol/L)	467	50 – 98
Na (mmol/L)	135	136-145
K (mmol/L)	5.6	3.5-5.1
Cl (mmol/L)	105	98-107
Bicarbonate (mmol/L)	17	22-29
Calcium (mmol/L)	2.22	2.15-2.50
C-reactive protein (CRP) (mg/L)	1.8	0-5.0
Amylase U/L	90	13-53
Lipase	88	13-60
Phosphate (mmol/L)	2.43	0.8-1.5
Parathyroid hormone (pg/ml)	292	15-65

**Table 2:** Autoimmune workup

Detail	Result
ANA	Positive
ANCA	Negative
Anti-MPO Ab	Negative
Anti-PR3 Ab	Negative
Anti-dsDNA	Negative
Anti-Jo-1	Negative
Anti-La	Negative
Anti-Ro	Positive
Anti-RNP	Negative
Anti-Scl-70	Negative
Anti-Sm	Negative
Anti-GBM	Positive

The patient was managed as a case of anti-GBM disease and was started on intravenous methylprednisolone 500 mg once daily for four days in addition to cyclophosphamide 100 mg orally daily and plasma exchange daily with observation of her kidney function. After 12 sessions of plasma exchange and two weeks of cyclophosphamide. Serum Cr improved to around 300 µmol/l and serum anti-GBM antibody became negative. The patient was then discharged on a tapering dose of oral steroids and oral cyclophosphamide. One month after discharge, the patient was followed in the nephrology clinic and showed stable serum creatinine.

## Discussion

Anti-GBM disease is a rare, well-characterized cause of glomerulonephritis. It is defined by the presence of autoantibodies directed at specific antigenic targets (Collagen alpha-3 (IV) chain) within the glomerular and/or pulmonary basement membrane and present with clinical features of rapidly progressive glomerulonephritis. Between 25 and 60 percent present with concomitant alveolar hemorrhage, and a small proportion of patients present with isolated pulmonary findings [2].

Anti-GBM disease is now classified as small vessel vasculitis caused by in situ immune complex formation; the diagnosis relies on the detection of anti-GBM in tissues or circulation in conjunction with alveolar or glomerular disease [3]. It has been suggested that the level of anti-GBM antibodies correlates with the long-term prognosis [4]. Type IV collagen of the glomerular basement membrane (GBM), an important component of the blood filtration barrier, is the target of pathogenic antibodies in anti-GBM antibody nephritis [5].

A kidney biopsy usually needs to be done to confirm the diagnosis, give an idea about the chronicity and disease activity, and exclude other causes. It typically shows crescentic glomerulonephritis with immunofluorescence microscopy, which demonstrates the finding of linear deposition of IgG along the glomerular capillaries and occasionally the distal tubules. In reported cases, the antibody may be IgA or immunoglobulin M (IgM) [6].

Anti-GBM disease can present with other variants, including patients with double-positive anti-GBM and ANCA antibodies, where overlap features of both diseases can present [7]. A rare variant of the anti-GBM disease, described as "atypical anti-GBM nephritis," has been reported where no circulating anti-GBM antibodies were found but kidney biopsy demonstrated bright, linear IgG deposition along the GBM but without features of crescentic glomerulonephritis [8]. Other forms of anti-GBM have been reported including anti-GBM disease associated with membranous nephropathy and anti-GBM after kidney transplantation for Alport syndrome [9-10].

A novel variant has been reported in a case series that investigated four patients with circulating anti-GBM antibodies, with focal necrotizing crescentic glomerulonephritis but no linear anti-GBM antibody deposition on immunohistochemistry. Three out of four patients were also ANCA positive. Serum from the patients showed linear binding to primate glomeruli by indirect immunofluorescence in two of the cases tested [1].

We report a case of a middle-aged female who presented with rapidly progressive glomerulonephritis and was found to have a high titer of anti-GBM antibody with kidney biopsy finding of crescentic glomerulonephritis without typical linear basement membrane staining. The patient was managed with intravenous methylprednisolone, cyclophosphamide, and plasmapheresis, which led to an improvement in her kidney function along with a decline in her anti-GBM antibody titer. In our case, we attribute the presence of a high anti-GBM titer to crescentic glomerulonephritis in the absence of linear deposition along the basement membrane, which may carry similarity to the novel variant of anti-GBM disease described in the case series [1].

Another pitch to explain the absence of linear deposition along the basement membrane in our case is that the staining was done on a paraffin-embedded sample rather than on a fresh sample, which carries false-negative results [11]. To confirm this theory, a repeat kidney biopsy with immunostaining on a fresh sample could be done. However, since the patient responded to treatment with stable kidney parameters, there was no clinical justification to repeat it.

Another argument is the possibility of the false positivity of serum anti-GBM, although rarely reported [12], along with another cause of crescentic glomerulonephrites, such as ANCA-negative vasculitis, may be present. Unfortunately, anti-GBM was not repeated to confirm its positivity before starting treatment, as we wanted to start treating the patient as early as possible to save the kidney before further damage.

## Conclusions

Anti-GBM disease can vary in its presentation, and positive anti-GBM without typical linear staining may represent a new variant. Thus, physicians would need to keep this in mind when formulating the diagnosis. Furthermore, we recommend that the immunostaining be done on a fresh sample to avoid false-negative results and to repeat serum anti-GBM antibody before starting treatment, especially when there are no supporting histopathological changes.
